# Unmasking Malignancy in Dense Breast Tissue (American College of Radiology Types C and D): A Biopsy-Proven Analysis of Digital Breast Tomosynthesis Performance, Clinical Decision Dynamics, and Correlation with Histological Grade and Molecular Subtypes

**DOI:** 10.3390/diagnostics16152321

**Published:** 2026-07-24

**Authors:** Abdulkadir Eren, Emrah Karatay, Ferhat Ozden

**Affiliations:** 1 Radiology, Istanbul Medipol University, Medipol Mega University Hospital, Istanbul 34214, Türkiye; aeren@medipol.edu.tr; 2 Radiology, Sultan 2. Abdulhamid Han Training and Research Hospital, Istanbul 34668, Türkiye; 3 Pathology, Istanbul Medipol University, Medipol Mega University Hospital, Istanbul 34214, Türkiye; ferhat.ozden@medipol.edu.tr

**Keywords:** digital breast tomosynthesis, breast density, ACR type C/D, architectural distortion, molecular subtypes, histological grade, clinical upgrade, sensitivity, biopsy-proven

## Abstract

**Background/Objectives:** This study aimed to evaluate the diagnostic performance and clinical decision-making impact of adding digital breast tomosynthesis (DBT) to two-dimensional digital mammography (2D DM) in a 100% biopsy-proven cohort of women with dense breast parenchyma (ACR types C and D), focusing on the relationship between tumor biology and radiologic conspicuity. **Methods:** This retrospective study analyzed 123 women (mean age 46.7 ± 9.9 years) with ACR type C (n = 67) or D (n = 56) breast density who underwent sequential 2D DM and DBT imaging, all with definitive histopathology (malignant, n = 78; high-risk, n = 8; benign, n = 37). A single expert radiologist performed independent readings of 2D DM alone and then the combined 2D DM + DBT dataset after a 4-week washout. Outcomes included sensitivity, specificity, PPV/NPV, lesion visibility (0–5 scale), and BI-RADS upgrade rate. **Results:** 2D DM + DBT significantly outperformed 2D DM alone in sensitivity (97.4% vs. 69.2%; *p* < 0.000001) and NPV (93.1% vs. 57.1%), with AUC improving from 0.727 to 0.922 (*p* < 0.001). Mean lesion visibility increased from 2.94 ± 1.19 to 4.52 ± 0.78 (*p* < 0.0001), with the largest gains for architectural distortion (100% sensitivity) and, notably, for Grade 1 tumors (delta +1.90), which reached the highest DBT visibility of any grade. Major BI-RADS upgrades occurred in 27 cases (22.0%; upgrade PPV 88.9%). **Conclusions:** In this biopsy-enriched ACR C/D cohort, adding DBT to 2D DM was diagnostically superior across all metrics. The disproportionate visibility gain for Grade 1 tumors is a hypothesis-generating finding relevant to the overdiagnosis debate, not proof that DBT reduces overdiagnosis. These findings support DBT as a valuable adjunct to 2D DM in dense breasts, pending prospective, multi-reader validation in unselected populations.

## 1. Introduction

The radiological evaluation of women with dense breast parenchyma (American College of Radiology [ACR] types C and D) represents one of the most clinically consequential challenges in contemporary oncological imaging. Breast density is a multifaceted problem: it is an independent risk factor for the development of breast carcinoma [1,2] while simultaneously acting as a physical mask that renders tumors invisible on conventional two-dimensional (2D) digital mammography (DM). This “masking effect” arises because both fibroglandular tissue and breast carcinomas share similar X-ray attenuation coefficients, creating a “white-out” background that obscures even large invasive lesions [2,3]. In most extremely dense (ACR D) breasts, 2D DM sensitivity has been reported to fall as low as 50–60%, rendering it diagnostically unreliable as a standalone modality [2].

Digital breast tomosynthesis (DBT) addresses this fundamental limitation by acquiring multiple low-dose X-ray projections at different angles and reconstructing them into quasi-three-dimensional (3D) thin slices (typically 1 mm). This technology allows the radiologist to “peel back” overlapping layers of normal fibroglandular tissue, exposing the structural irregularities—spiculated masses, architectural distortions, and focal asymmetries—that signify early malignancy [4]. Unlike supplemental ultrasound or MRI, DBT adds minimal examination time, does not require contrast administration, and is performed on the same platform as the standard mammogram, making it operationally viable for routine clinical deployment [5].

Large-scale population-based screening trials (e.g., Oslo, STORM, and TOSYMA) have established the foundational evidence for DBT, consistently demonstrating increased cancer detection rates compared to 2D DM alone [6,7,8].

A particularly underexplored dimension is the relationship between DBT performance and tumor biology. Different molecular subtypes of breast cancer present with distinct morphological signatures: Luminal A carcinomas tend to be slow-growing and may present as subtle architectural distortions, whereas triple-negative and Luminal B carcinomas often present as circumscribed masses that can mimic benign lesions on 2D projections [9]. Whether DBT provides uniform diagnostic benefit across the full biological spectrum—from indolent Grade 1 to aggressive Grade 3 tumors—remains largely uncharacterized. This distinction has direct implications for the ongoing debate about overdiagnosis: critics argue that DBT primarily detects slow-growing, clinically insignificant tumors [10], while proponents contend that it uncovers genuinely occult, biologically significant cancers that would otherwise progress to interval cancer status.

Furthermore, architectural distortion (AD)—the third most common radiological sign of non-palpable breast cancer—poses a particularly acute diagnostic challenge in dense breasts. Its radial, spiculated character is geometrically indistinguishable from overlapping fibroglandular tissue on 2D projections, making it one of the most frequently missed diagnoses on standard mammography [11,12]. DBT’s slice-based reconstruction is theorized to be uniquely suited to its detection, yet the magnitude of this advantage in a biopsy-confirmed cohort has not been rigorously quantified.

This study was designed to address these evidence gaps through a 100% biopsy-proven analysis of 123 women with ACR C/D breast density. Our primary aim was to quantify the diagnostic superiority of DBT over 2D DM in terms of sensitivity, specificity, and area under the ROC curve. Secondary aims included (1) characterizing the magnitude of DBT’s visibility advantage by lesion morphology, with particular focus on architectural distortion; (2) determining the rate and clinical significance of major BI-RADS upgrades and their pathological outcomes; and (3) investigating the novel hypothesis that histological grade and Ki67 proliferation index correlate inversely with DBT visibility gain—providing direct, biopsy-confirmed evidence to inform the overdiagnosis debate.

## 2. Materials and Methods

### 2.1. Study Design and Ethics

This retrospective, single-center diagnostic accuracy study was conducted at a tertiary breast imaging referral center. This study was conducted in accordance with the Declaration of Helsinki (1975, revised in 2013), and the protocol was approved by the Non-Interventional Clinical Research Ethics Committee of Istanbul Medipol University (Approval No: 999; Approval Date: 25 May 2026). Informed consent for publication was obtained from all identifiable human participants. All procedures adhered to the STARD 2015 (Standards for Reporting of Diagnostic Accuracy Studies) reporting guidelines.

### 2.2. Patient Population

From a consecutive clinical registry spanning 2023–2026, 123 women (age range, 27–82 years; mean ± SD, 46.7 ± 9.9 years) with ACR type C (heterogeneously dense; n = 67, 54.5%) or type D (extremely dense; n = 56, 45.5%) breast parenchyma were retrospectively identified [1,2]. Breast density was assessed visually according to the ACR BI-RADS 5th edition lexicon by the interpreting radiologist; no automated volumetric software was used. The defining methodological feature of this cohort is that every patient underwent either core needle biopsy or surgical excision, yielding a definitive histopathological reference standard for 100% of included cases. Patients were excluded if they had prior ipsilateral breast surgery, breast implants, or a personal history of breast malignancy to avoid diagnostic confounding. Patient and lesion characteristics are summarized in Table 1.

### 2.3. Reference Standard and Histopathological Classification

All biopsy specimens were reviewed by a dedicated breast pathologist blinded to imaging findings. Pathological outcomes were categorized as (1) Malignant [n = 78, 63.4%], comprising invasive carcinomas and ductal carcinoma in situ; (2) High-Risk [n = 8, 6.5%], encompassing lesions with recognized malignant upgrade potential, including radial scars, atypical ductal hyperplasia (ADH), and intraductal papillomas with atypia; and (3) Benign [n = 37, 30.1%], including fibroadenomas, adenosis, and usual ductal hyperplasia. For the primary diagnostic performance analysis, the High-Risk and Malignant groups were combined as the positive reference, consistent with established methodology for biopsy-outcome studies, as both categories necessitate mandatory surgical excision or rigorous clinical intervention. We acknowledge that high-risk lesions (radial scar, ADH, and atypical papilloma) are not oncologically equivalent to invasive carcinoma or DCIS; because only 8 of 123 cases (6.5%) fell into this category, its inclusion in the positive reference is unlikely to have materially altered the primary performance estimates, but this should be considered when interpreting the pooled results.

Molecular subtype classification followed the St. Gallen 2023 consensus criteria and was performed via standardized immunohistochemistry for estrogen receptor (ER), progesterone receptor (PR), HER2/neu (HER2), and Ki67 proliferation index: Luminal A (ER+ and/or PR+, HER2−, and Ki67 < 20%); Luminal B (ER+ and/or PR+, HER2+ or Ki67 ≥ 20%); HER2-enriched (ER−, PR−, and HER2+); and triple-negative (ER−, PR−, and HER2−). HER2 status was confirmed by fluorescence in situ hybridization (FISH) in all equivocal (2+) IHC cases. Biopsy was not based on mammography/DBT findings alone in all cases: a documented indication for biopsy independent of, or in addition to, the mammographic/DBT finding was present in 28 of 123 patients (22.8%), most commonly a suspicious finding on supplemental ultrasound (21/123, 17.1%); other documented indications included suspicious findings on MRI (n = 1), interval increase in lesion size (n = 3), axillary lymphadenopathy (n = 1), known pathogenic germline variant (n = 1), and a newly palpable finding (n = 1). In the remaining 95 patients (77.2%), biopsy was prompted directly by the mammography/DBT finding itself.

### 2.4. Imaging Protocol

All patients underwent standard two-view (craniocaudal [CC] and mediolateral oblique [MLO]) two-dimensional digital mammography (2D DM) followed immediately by DBT acquisition on a single high-resolution integrated system (Siemens Mammomat Revelation, Siemens Healthineers, Erlangen, Germany). DBT projections were acquired at 15 degrees per tube movement (25 total exposures) and reconstructed into 1 mm thick slices using the system’s iterative reconstruction algorithm [4]. The mean glandular dose per view was 1.24 ± 0.40 mGy for 2D DM and 2.25 ± 0.67 mGy for DBT, both of which were maintained strictly within international regulatory limits. The reported mean glandular dose values for both 2D DM (1.24 ± 0.40 mGy) and DBT (2.25 ± 0.67 mGy) are well within the diagnostic reference levels (DRLs) established by the American College of Radiology (ACR) and the European Society of Breast Imaging (EUSOBI), confirming that our imaging protocol maintains optimal radiation safety standards for dense breast screening. All images were interpreted on a dedicated high-resolution 5-megapixel diagnostic workstation (Siemens Syngo.via, Version 5.0) with calibrated ambient lighting.

### 2.5. Image Interpretation Protocol

A single breast radiologist with more than 15 years of subspecialty experience performed all image evaluations. To minimize recall bias, the two reading sessions were separated by a mandatory 4-week washout period during which all clinical and pathological information was withheld: Session 1 consisted of 2D DM images alone; Session 2 consisted of the combined 2D DM + DBT dataset reviewed de novo.

Lesion visibility was scored using a 6-point ordinal Likert scale developed for this analysis: 0, not visible (completely masked by dense tissue); 1, barely perceptible; 2, subtle but identifiable with effort; 3, moderately conspicuous; 4, clearly visible with well-defined characteristics; 5, excellent conspicuity with complete lesion delineation. As all scoring was performed by a single reader, this scale should be regarded as a semi-quantitative internal tool rather than an externally validated instrument, and intra- or inter-reader reliability could not be assessed in the present design. Throughout this manuscript, unless otherwise specified, “DBT” and “DBT visibility/sensitivity” refer to the second reading session, which consisted of the combined 2D DM + DBT dataset rather than DBT interpreted in isolation; this shorthand is used for readability and is not intended to imply that DBT was evaluated as a stand-alone modality. Lesion morphology was categorized as mass (type 1), architectural distortion (type 2), asymmetry (type 3), or combinations thereof. Pure microcalcifications were excluded from morphological analysis by study design. All findings were categorized according to the ACR BI-RADS 5th Edition lexicon.

### 2.6. Definition of Clinical Decision Categories

A major upgrade was defined as a management-changing shift in which a lesion assigned BI-RADS 1, 2, or 3 (no immediate biopsy recommended) on 2D DM alone was reclassified to BI-RADS 4A, 4B, 4C, or 5 (biopsy recommended) after DBT review. A major downgrade was defined as a shift from BI-RADS 4 or higher on 2D DM to BI-RADS 3 or lower following DBT. Total decision changes encompassed any inter-session BI-RADS category shift, including upgrades within the BI-RADS 4 spectrum (e.g., 4A to 4B). The upgrade positive predictive value (PPV) was calculated as the proportion of major upgrades confirmed as malignant or high-risk on final histopathology.

### 2.7. Statistical Analysis

All statistical analyses were performed using SPSS Version 26.0 (IBM Corp., Armonk, NY, USA) and Python 3.12 (scipy v1.13, statsmodels v0.14). Sensitivity, specificity, positive predictive value (PPV), and negative predictive value (NPV) were calculated for 2D DM and 2D + DBT using the biopsy result as the gold standard. Wilson score method 95% confidence intervals (CIs) were computed for all proportional estimates. Paired sensitivity comparison was performed using McNemar’s exact binomial test. The Wilcoxon signed-rank test was used to compare paired visibility scores between sessions. Differences in visibility gain (delta scores) between lesion morphological subtypes were evaluated with the Mann–Whitney U test. Monotonic associations between histological grade or Ki67 and visibility scores were assessed by Spearman’s rank correlation coefficient. Area under the receiver operating characteristic curve (AUC) was estimated with the trapezoidal method and compared across modalities using the DeLong method for correlated ROC curves. For small subgroups (n < 15), results are reported with 95% CIs and interpreted cautiously; Fisher’s exact test was substituted for chi-square where expected cell counts fell below 5. A two-tailed *p*-value less than 0.05 was considered statistically significant. A formal a priori sample size calculation was not performed due to the retrospective registry design; however, a post hoc power analysis indicated that the observed cohort (n = 123, with 86 positive cases) provided greater than 90% power to detect the observed 28-percentage-point sensitivity difference at alpha = 0.05 using McNemar’s test, supporting the adequacy of the sample for the primary endpoint.

## 3. Results

### 3.1. Overall Diagnostic Performance

**Figure 1:**
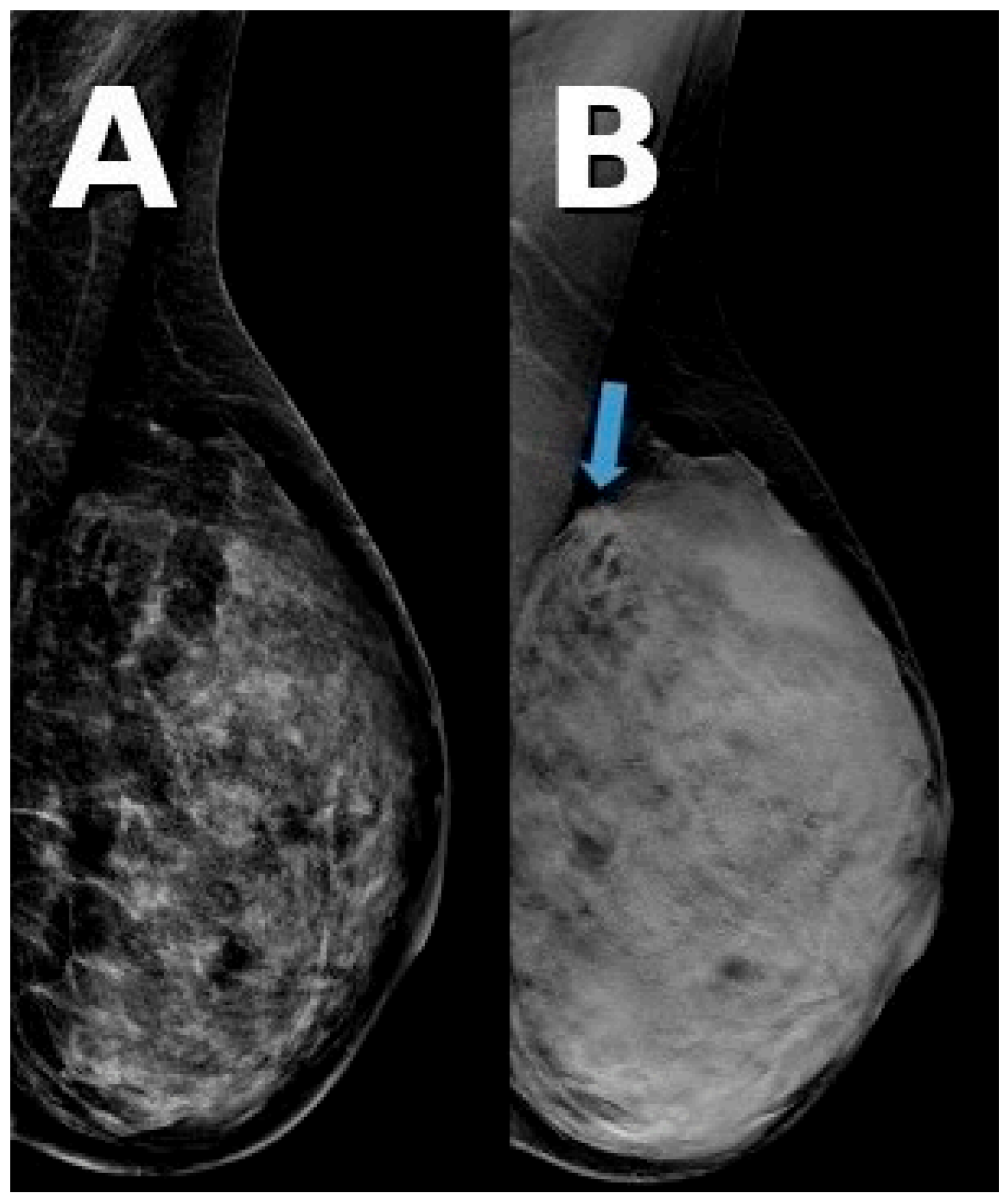
Comparison of 2D DM and DBT in a 40-year-old patient with extremely dense breasts (ACR Type D): (**A**) 2D DM shows no suspicious findings (Visibility Score 1, BI-RADS 2). (**B**) The corresponding DBT slice clearly unmasks a mass with irregular, spiculated margins causing parenchymal distortion (arrow), resulting in an upgrade to BI-RADS 4C (Visibility Score 4). Histopathology revealed a Grade 2 invasive ductal carcinoma (Luminal B: ER+, PR+, HER2−, Ki-67 15%).

The addition of DBT to 2D DM produced clinically and statistically significant improvements across all primary performance metrics (Table 2). Sensitivity increased from 69.2% (95% CI, 58.3–78.4%) with 2D DM to 97.4% (95% CI, 91.1–99.3%) with 2D + DBT—an absolute gain of 28.2 percentage points (*p* < 0.001) [6,7,13]. In the paired malignant case comparison, 22 tumors were correctly identified exclusively by DBT, and zero tumors were detected exclusively by 2D DM (McNemar exact *p* < 0.000001), demonstrating a strictly unidirectional diagnostic advantage. NPV improved from 57.1% to 93.1%, substantially reducing the risk of false reassurance in the dense breast setting [3]. AUC increased from 0.727 to 0.922 (*p* < 0.001, DeLong method). Specificity decreased modestly from 71.1% to 60.0% (*p* = 0.21, non-significant), attributable in part to the detection of additional actionable findings subsequently confirmed as high-risk or malignant [14,15]; however, 3 of 27 major upgrades (11.1%) represented false-positive biopsies, representing a real patient-level cost that must be weighed against the substantial sensitivity benefit in clinical decision-making (Figure 1).

### 3.2. Lesion Visibility and Morphological Analysis

**Figure 2:**
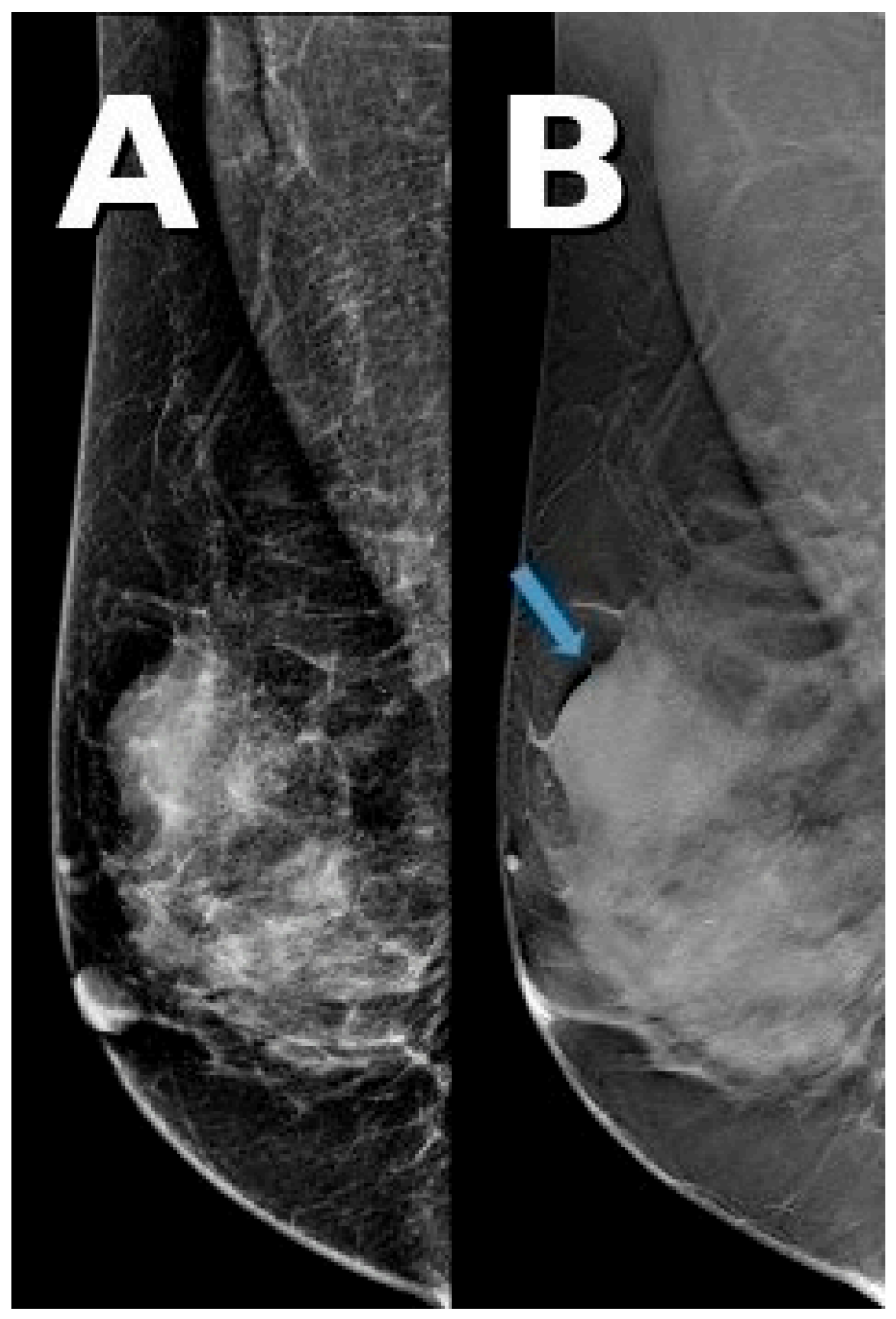
Mediolateral oblique (MLO) view comparison in a 44-year-old patient with extremely dense breasts (ACR Type D): (**A**) 2D DM demonstrates a partially visible nodule with adequate conspicuity (Visibility Score 4); however, insufficient marginal characterization on the two-dimensional projection precluded definitive classification, necessitating an incomplete assessment (BI-RADS 0) and additional imaging. (**B**) The corresponding DBT slice resolves this diagnostic uncertainty by clearly delineating an irregular mass with indistinct and spiculated margins (arrow), enabling definitive reclassification to BI-RADS 4A with excellent visibility (Score 5). Histopathology confirmed an aggressive Grade 3 invasive ductal carcinoma (Luminal B: ER+, PR−, HER2+, Ki-67 80%). This case illustrates that lesion detection does not preclude diagnostic uncertainty on 2D DM and that DBT provides the marginal characterization necessary to convert an incomplete assessment into an actionable diagnosis.

Mean visibility score increased from 2.94 ± 1.19 on 2D DM to 4.52 ± 0.78 on DBT (Wilcoxon W = 0.0, *p* < 0.0001). This improvement was not uniform across morphological subtypes. Architectural distortion (AD)-containing cases—31 pure AD and 34 mixed mass-AD morphologies (n = 65 total)—showed the greatest absolute visibility gain (delta = 1.95), nearly double that of mass-only lesions (delta = 1.13; Mann–Whitney *p* < 0.0001). In the malignant subset with AD morphology (n = 52), DBT achieved 100% sensitivity compared to 69.2% for 2D DM. Of 8 cases assigned BI-RADS 0 (incomplete) on 2D DM, DBT resolved all 8: 7 were reclassified to BI-RADS 4A–5 (all histopathologically confirmed malignant), and 1 was appropriately downgraded to BI-RADS 3 (benign on biopsy) (Figure 2).

### 3.3. Histological Grade, Ki67, and the Early-Detection Paradox

A clinically meaningful inverse pattern was observed between histological grade and DBT visibility gain (Table 3). Grade 1 tumors exhibited the lowest mean visibility on 2D DM (2.75 ± 1.33) yet achieved the highest mean visibility on DBT (4.65 ± 0.59, delta = +1.90). Grade 3 tumors were more conspicuous on 2D DM (3.38 ± 0.92) and demonstrated a comparatively smaller but still significant gain on DBT (4.52 ± 0.60, delta = +1.14). Spearman’s correlation between histological grade and 2D DM visibility trended positive (r = +0.208, *p* = 0.071), while the corresponding correlation for DBT visibility was negligible (r = −0.084, *p* = 0.471), indicating that DBT effectively equalizes conspicuity across the tumor grade spectrum.

**Figure 3:**
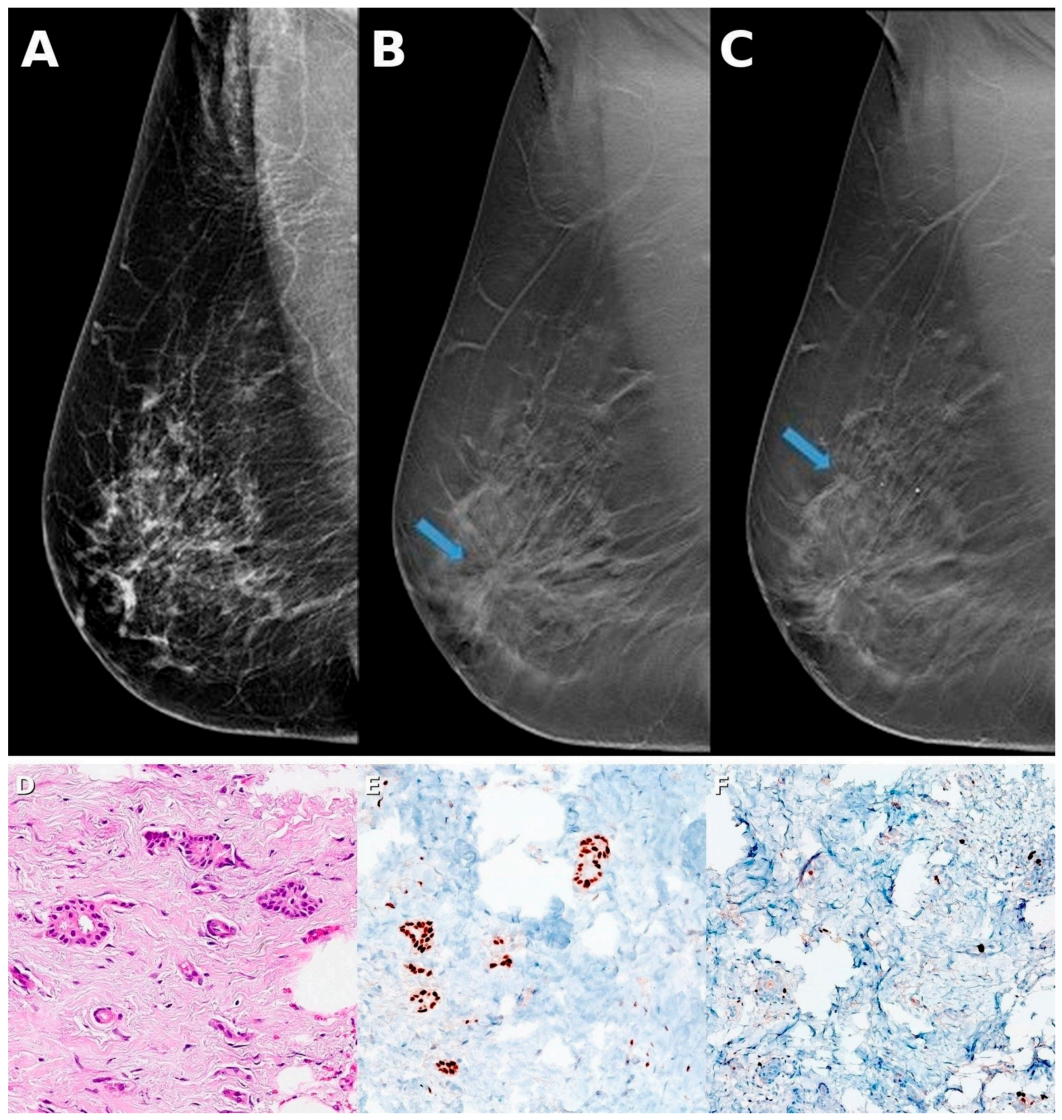
Radio-pathologic correlation of an occult Grade 1 invasive ductal carcinoma in a 54-year-old patient with heterogeneously dense breast parenchyma (ACR Type C): (**A**) 2D DM evaluation demonstrates dense fibroglandular tissue completely masking the underlying lesion (Visibility Score 1), resulting in an initial BI-RADS 2 classification. (**B**) The corresponding DBT slice unmasks a distinct architectural distortion (arrow) with clear visibility (Visibility Score 4), leading to a major clinical upgrade to BI-RADS 5. (**C**) A separate DBT slice reveals a second occult focus of architectural distortion (arrow), similarly masked on 2D DM, consistent with multifocal disease. (**D**) Hematoxylin and eosin (H&E) staining (×25) confirms a well-differentiated (Grade 1) carcinoma accompanied by a pronounced desmoplastic stromal reaction; this fibrotic response explains the architectural distortion uniquely captured by DBT. (**E**) Immunohistochemistry (IHC) for estrogen receptor (ER) (×20) demonstrates strong ER positivity, characteristic of a biologically indolent Luminal A phenotype that typically grows via displacement rather than rapid infiltration. (**F**) Ki-67 immunohistochemistry (×18) reveals a low proliferation index (10%), further supporting the Luminal A classification and the indolent biological behavior of this tumor.

For Ki67, tumors with low proliferative index (<20%) showed numerically greater DBT visibility (4.63 ± 0.63) than high-Ki67 tumors (≥20%; 4.45 ± 0.61), consistent with the grade findings, though this difference did not reach statistical significance (Mann–Whitney *p* = 0.151; Spearman r = −0.142, *p* = 0.221). Multifocal involvement was documented in one Grade 1 case, in which a second occult focus of architectural distortion—invisible on 2D DM—was unmasked exclusively by DBT, further illustrating the modality’s capacity to delineate the full extent of low-grade disease (Figure 3).

### 3.4. Molecular Subtype Sensitivity

**Figure 4:**
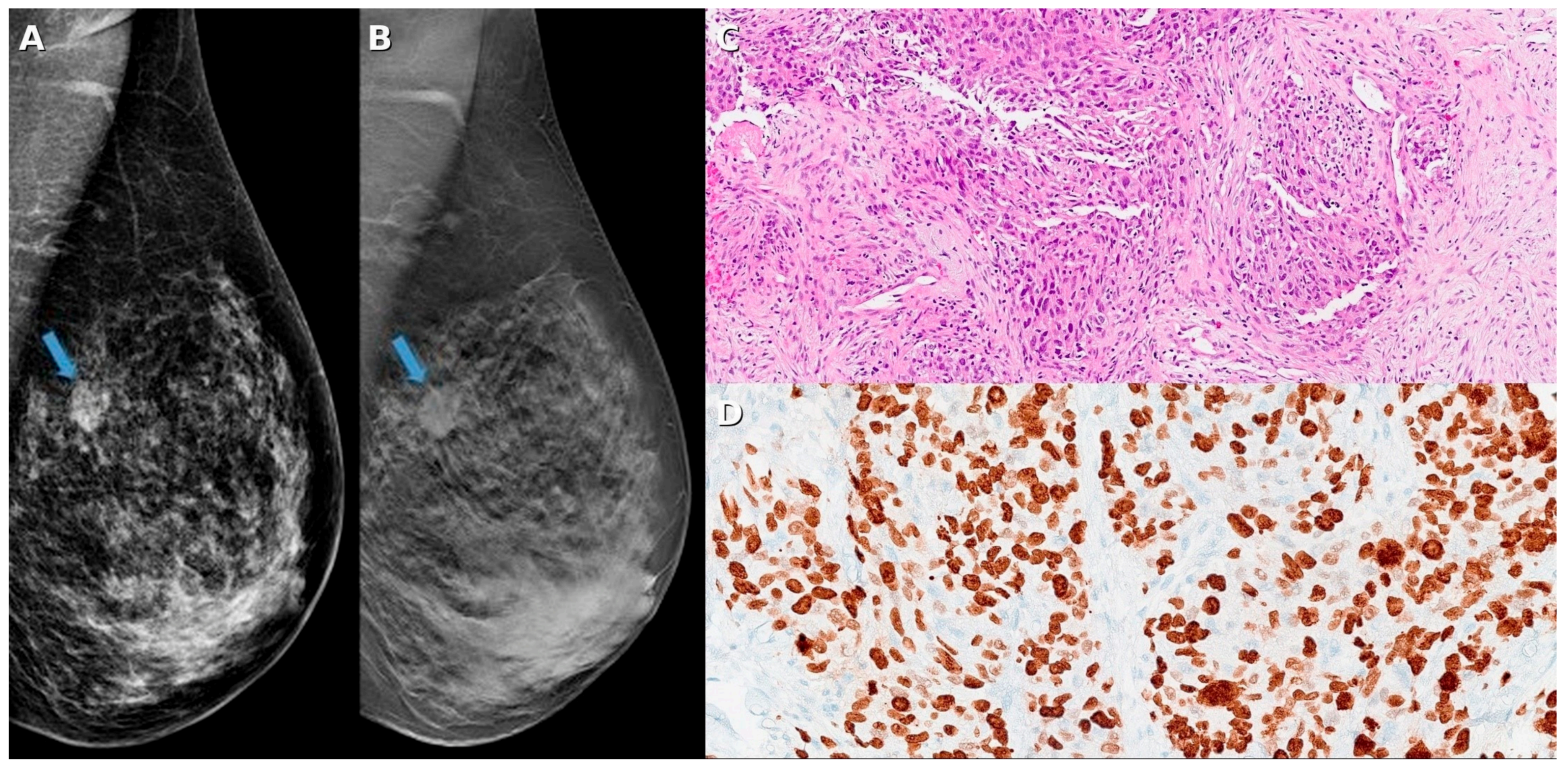
Radio-pathologic correlation of a triple-negative breast cancer (TNBC) in a 49-year-old woman with extremely dense breast parenchyma (ACR Type D): (**A**) Two-dimensional digital mammography (2D DM, left breast MLO view) demonstrates a partially obscured mass with seemingly circumscribed margins (arrow), initially categorized as BI-RADS 3. (**B**) The corresponding digital breast tomosynthesis (DBT) slice reveals spiculated margins and lobulated contours (arrow), warranting reclassification to BI-RADS 5. (**C**) Hematoxylin and eosin (H&E) staining (×20) confirms Grade 3 invasive ductal carcinoma (IDC) with marked nuclear pleomorphism and atypical mitoses. (**D**) Ki-67 immunohistochemistry (IHC, ×40) demonstrates a proliferation index of 80%, consistent with the aggressive TNBC phenotype (ER−, PR−, and HER2− confirmed by FISH).

DBT provided the largest sensitivity gains in subtypes most susceptible to masking on 2D DM. Luminal B tumors (n = 38)—often presenting as ill-defined masses or subtle architectural distortions without microcalcifications—showed sensitivity increasing from 68.4% (95% CI, 52.5–80.9%) to 97.4% (95% CI, 86.5–99.5%) with DBT. Triple-negative carcinomas (n = 10), which characteristically present as circumscribed masses that mimic benign lesions on 2D projections, reached 100% DBT sensitivity (95% CI, 72.2–100%); this result should be interpreted with caution given the small subgroup size and correspondingly wide confidence interval. Luminal A tumors (n = 26) similarly improved from 69.2% to 96.2%. The two HER2-enriched cases achieved 100% sensitivity with both modalities and are reported descriptively only (Figure 4).

### 3.5. Clinical Decision Dynamics

**Figure 5:**
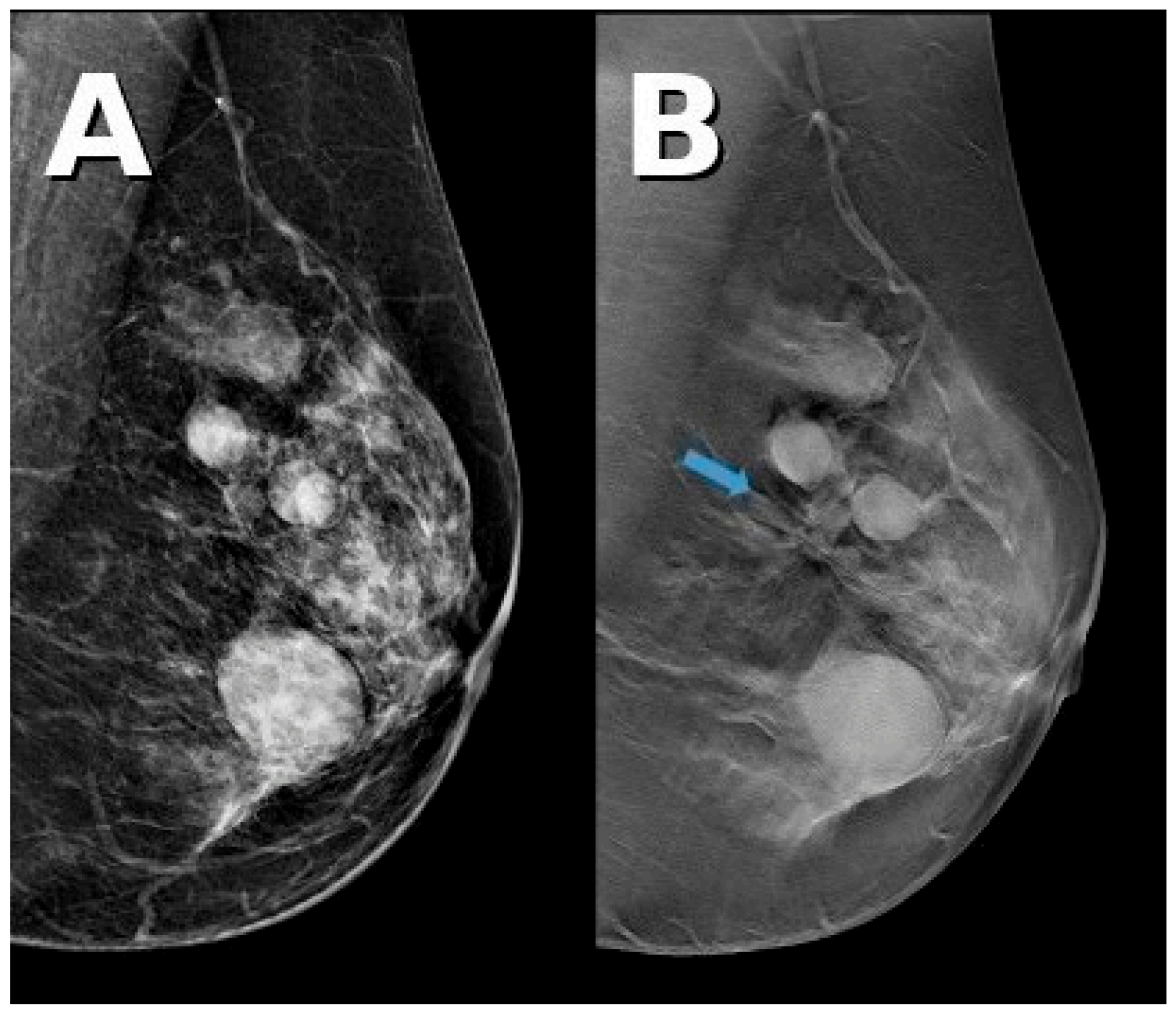
Mediolateral oblique (MLO) view comparison of the left breast in a 44-year-old patient with heterogeneously dense parenchyma (ACR Type C): (**A**) Initial 2D DM reading shows dense parenchymal patterns completely masking the lesion, interpreted as BI-RADS 2 (Visibility Score 1). (**B**) The corresponding DBT slice (arrow) unmasks the architectural distortion with excellent visibility (Visibility Score 5), resulting in a major upgrade to BI-RADS 4C. Histopathology confirmed a high-risk lesion, specifically a radial scar.

**Figure 6:**
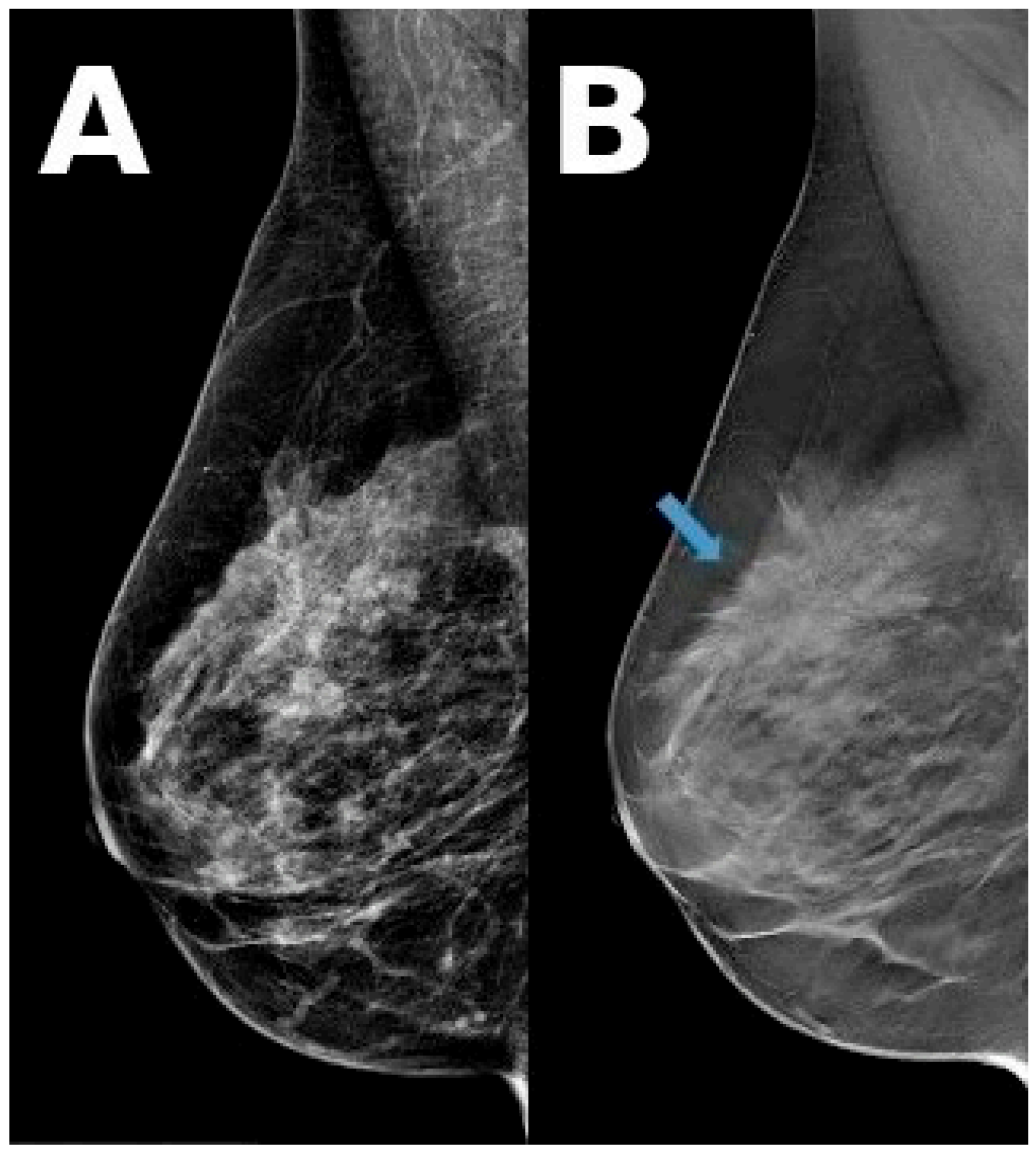
Mediolateral oblique (MLO) view comparison of the left breast in a 45-year-old patient with heterogeneously dense breast parenchyma (ACR Type C): (**A**) The lesion is completely obscured by dense fibroglandular tissue on 2D DM, categorized as BI-RADS 2 (Visibility Score 1). (**B**) DBT evaluation unmasks the underlying structural abnormality, providing good visibility (Visibility Score 4) and leading to a clinical upgrade to BI-RADS 4B. Histopathological examination revealed a benign diagnostic challenge, confirmed as sclerosing adenosis.

A total of 27 major upgrades (22.0% of the total cohort) occurred, each representing a cancer or high-risk lesion that 2D DM would have deferred to follow-up or dismissed as probably benign (Table 4). Of these, 22 (81.5%) were confirmed malignant and 2 (7.4%) were high-risk on pathology, yielding an upgrade PPV of 88.9%. Three cases (11.1%) were false-positive upgrades (benign histology). No major downgrades occurred: DBT did not reclassify any BI-RADS 4+ finding to a follow-up category, confirming that tomosynthesis in the hands of an experienced reader does not introduce dangerous underclassification. The total decision change rate (any inter-session BI-RADS shift) was 61.8% (n = 76), reflecting the broad impact of DBT on diagnostic categorization in the dense-breast setting. (Figure 5 and Figure 6).

### 3.6. Surgical and Oncologic Characteristics in the Definitive-Surgery Subset

Tumor staging, surgical pathology, and treatment-related variables were retrieved from institutional oncology follow-up records for the subset of malignant cases in which definitive surgery was performed at our own institution (49/78, 62.8%); the remaining 29 patients were referred for surgery at another center or lost to follow-up, and these variables could not be retrieved for them.

In this subset (Table 5), pathological tumor stage was T1 in 22 cases (44.9%), T2 in 15 (30.6%), T3 in 3 (6.1%), and T4 in 1 (2.0%); 8 patients (16.3%) who received neoadjuvant chemotherapy achieved a pathologic complete response (ypT0). Nodal status was negative (N0) in 26 cases (53.1%) and positive in 21 (42.9%; N1–N3). Multifocal disease was present in 15 patients (30.6%) and multicentric disease in 3 (6.1%); lymphovascular invasion was identified in 1 patient (2.0%). Definitive surgery consisted of mastectomy in 31 cases (63.3%) and breast-conserving surgery in 18 (36.7%). Twenty-seven patients (55.1%) received neoadjuvant chemotherapy, of whom 8/24 (33.3%) with a recorded Miller–Payne grade achieved a Grade 5 (complete) pathologic response. The interval from biopsy to definitive surgery was, as expected, bimodal: a median of 22 days (range 6–36) for patients proceeding directly to surgery, versus a median of 175 days (range 60–222) for those receiving neoadjuvant chemotherapy before surgery, consistent with standard neoadjuvant treatment timelines.

These data indicate that this cohort was not restricted to early, node-negative disease: node-positive, multifocal, and locally advanced (T3/T4) tumors were well represented, and a substantial minority underwent neoadjuvant systemic therapy. However, because complete surgical-pathology data were retrievable for only 62.8% of malignant cases, and because individual DBT-detected findings were not systematically linked to specific surgical decisions in this registry, we are unable to determine whether DBT’s incremental lesion detection directly altered surgical planning, margin strategy, or the choice between breast-conserving surgery and mastectomy in individual cases; this remains an important question for future prospective research incorporating multidisciplinary tumor board data.

## 4. Discussion

This biopsy-proven study of 123 women with ACR C/D breast density provides granular evidence that DBT is a valuable adjunct to 2D DM that may meaningfully improve diagnostic characterization and lesion detection in this high-risk, biopsy-enriched population; because the cohort was selected on the basis of biopsy, these findings require confirmation in unselected populations before broader practice recommendations can be made. The 28.2 percentage-point sensitivity gain (69.2% to 97.4%) with McNemar *p* < 0.000001—derived from a 100% biopsy-confirmed cohort—represents a statistically robust paired comparison, extending findings from landmark screening trials (Oslo, STORM) to the definitively characterized biopsy-outcome setting [1,2,6,7].

DBT’s 100% sensitivity for malignant architectural distortion (AD), versus 69.2% for 2D DM, was the most clinically urgent finding, with a significantly greater visibility gain for AD than for mass morphology (delta 1.95 vs. 1.13; *p* < 0.0001). AD’s spiculated, radial character is geometrically indistinguishable from overlapping fibroglandular tissue on two-dimensional summation images [11], but DBT’s slice-based reconstruction separates tissue planes and reveals the radiating lines of an invasive margin without background noise [4]. This aligns with and extends Partyka et al. [12], who found that DBT-detected AD occult on 2D DM carries a high PPV for malignancy.

The most scientifically novel finding of this study is the inverse relationship between histological grade and DBT visibility gain. Grade 1 tumors—biologically indolent, slow-growing—were the most occult on 2D DM (mean visibility 2.75) yet achieved the highest visibility on DBT (4.65), a paradox with a coherent biological explanation: low-grade invasive carcinomas frequently grow by displacement rather than infiltration, producing subtle architectural distortions and ill-defined non-calcified masses rather than the dense sclerotic tumors or extensive microcalcifications that render high-grade cancers relatively more conspicuous on 2D projections [3,9]. DBT’s plane-separation mechanism is uniquely suited to detect these subtle structural changes [4].

This finding is relevant to, though it does not resolve, the broader debate over whether increased detection represents overdiagnosis of indolent tumors [10]. That these Grade 1 tumors were essentially invisible on 2D DM is consistent with—but does not prove—the hypothesis that DBT identifies genuinely occult lesions rather than ones that would eventually surface on 2D DM; confirming this would require longitudinal follow-up, interval cancer rates, and outcome data beyond the scope of this retrospective design. The negligible correlation between grade and DBT visibility (Spearman r = −0.084, *p* = 0.471) is consistent with DBT equalizing detection across the tumor biology spectrum rather than selectively enriching for indolent or aggressive disease [8,16].

Luminal B and triple-negative carcinomas—the subtypes most often responsible for interval cancers diagnosed after initial false-negative imaging [17,18]—showed near-complete DBT sensitivity in our cohort (97.4% and 100%, respectively), versus approximately 68–70% on 2D DM; the 100% triple-negative figure warrants cautious interpretation given the small subgroup (n = 10; CI 72.2–100%). Triple-negative carcinomas often present as smooth, circumscribed masses radiologically indistinguishable from benign lesions on 2D projections [9], whereas DBT’s multiplanar reconstruction reveals marginal microlobulation and posterior contour irregularities—hallmarks of the invasive front—that are otherwise obscured by dense tissue on 2D [4,19].

The 88.9% upgrade PPV (24 of 27 upgrades confirmed malignant or high-risk) is exceptionally high relative to benchmark values in the DBT literature (typically 60–80%) [13,20] and reflects the strict operational definition of “major upgrade” applied in this study. Only 3 of 27 upgrades (11.1%) represented false-positive biopsies. Critically, the absence of major downgrades indicates that DBT in experienced hands does not introduce underestimation even as it resolves the false-positive asymmetries that inflate recall rates on 2D DM [21].

DBT visibility scores remained high in both ACR C (4.57) and ACR D (4.46) breasts, with no statistically significant difference (Mann–Whitney *p* = 0.259). This finding argues against the clinical concern that DBT performance degrades in extremely dense tissue [18] and supports a uniform recommendation for DBT across the full ACR C/D spectrum, rather than reserving it preferentially for ACR C patients [8,15].

This study did not evaluate ultrasound or MRI as primary diagnostic tools, and our findings should not be interpreted as evidence against the current, widely adopted recommendation to add supplemental ultrasound to mammography in dense breasts. To the contrary, a suspicious finding on supplemental ultrasound was the documented indication for biopsy in 21 of 123 patients (17.1%) in this cohort (Section 2.3), confirming that ultrasound identified clinically significant findings in a meaningful subset of cases in our own data. DBT and ultrasound reduce the masking effect of dense tissue through different mechanisms—structural plane separation versus acoustic tissue characterization—and are complementary rather than competing tools in clinical practice. Likewise, this study did not include MRI, and while DBT substantially narrows the sensitivity gap with 2D DM in dense breasts, we cannot conclude from these data whether DBT reduces the clinical need for MRI, particularly in patients at elevated hereditary or lifetime risk; this comparison would require a dedicated prospective study incorporating all three modalities.

Our results align with and expand upon recent investigations evaluating DBT performance in high-risk and histology-correlated cohorts. Over the past few years, several studies have consistently demonstrated that DBT significantly reduces the masking effect in dense parenchyma, particularly for architectural distortions [22]. However, while much of the recent literature focuses on broad screening metrics, our study contributes granular, biopsy-proven correlations between DBT visibility gain and intrinsic tumor biology to this body of evidence, specifically highlighting the paradoxical visibility of Grade 1 tumors.

Beyond human-reader performance, integrating artificial intelligence, deep learning, and radiomics with DBT datasets is a promising frontier: emerging evidence suggests such approaches can further enhance lesion stratification, reduce false-positive recalls, and potentially identify sub-visual imaging biomarkers linked to molecular subtype [23], further optimizing the diagnostic workflow for women with ACR C and D breast density.

Several limitations relate to study design and reader variability. First, the single-reader design limits formal assessment of inter-observer variability and represents the primary methodological constraint of this study; the performance metrics reported here reflect the capabilities of a highly experienced subspecialist and may not generalize to readers with less experience. Future multicenter studies should validate these findings across multiple reader experience levels and report inter-reader agreement (kappa). Second, the retrospective biopsy-proven design introduces enrichment bias: because all 123 patients underwent tissue sampling, the cohort is systematically enriched for clinically suspicious findings. In a prospective population-based screening setting—where the vast majority of examinations yield negative results—specificity, PPV, and NPV would be expected to differ substantially from the values reported here, and the findings should not be extrapolated directly to unselected screening populations. Furthermore, while our cohort demonstrated 100% sensitivity for both triple-negative carcinomas and architectural distortion-type malignancies, we acknowledge that these perfect detection rates are inherently sample-dependent. They likely represent the upper bound of DBT performance in a highly selected, biopsy-proven cohort rather than an absolute population metric. Third, small subgroup sizes for triple-negative (n = 10) and HER2-enriched (n = 2) cancers preclude definitive subtype-level conclusions, and confidence intervals for these groups are necessarily wide. Fourth, the washout period of 4 weeks between reading sessions minimizes but does not entirely eliminate recall bias; because the reading order was fixed (2D DM first, combined 2D DM + DBT second, rather than randomized or counterbalanced), the second session carried an inherent informational advantage, which future studies should address with a randomized or counterbalanced design.

Additional limitations relate to variables not systematically captured in this retrospective registry. The fifth limitation is that the absence of automated volumetric breast density software prevents correlation of quantitative fibroglandular volume with lesion visibility. Sixth, the visibility scale used in this study was developed for this analysis and scored by a single reader; it should be regarded as a semi-quantitative internal tool rather than an externally validated or reliability-tested instrument. Seventh, this registry did not systematically capture whether patients presented symptomatically, were recalled from screening, or were referred after an abnormal finding; this heterogeneity of clinical indication, combined with the malignancy rate exceeding 60% in this biopsy-proven cohort, further limits extrapolation of the reported sensitivity, specificity, PPV, and NPV to a screening or general diagnostic population. Eighth, as detailed in Section 3.6, tumor stage, nodal status, multifocality, lymphovascular invasion, and definitive surgical treatment were retrieved for the subset of malignant cases in which surgery was performed at our own institution (49/78, 62.8%) but were not available for the remainder of the cohort; moreover, individual DBT-detected findings were not systematically linked to specific surgical decisions in this registry, so the direct impact of DBT on surgical planning and patient outcomes remains an important area for future research. These limitations should guide the design of future prospective, multi-reader, randomized-order studies.

## 5. Conclusions

In this biopsy-enriched cohort of women with ACR C and D breast density, the addition of digital breast tomosynthesis to 2D mammography substantially improved diagnostic performance compared with 2D mammography alone. By increasing sensitivity from 69.2% to 97.4% and facilitating high-PPV biopsy upgrades in 22% of cases, DBT addition reduced the masking effect of dense parenchyma in this selected, biopsy-proven population. The novel finding that Grade 1, histologically indolent tumors show the greatest absolute visibility gain with DBT is a hypothesis-generating observation relevant to the overdiagnosis debate, consistent with—though not proof of—the possibility that these are genuinely occult lesions rather than lesions detected only marginally earlier; confirming this would require prospective follow-up and interval cancer data. The 100% sensitivity for architectural distortion-type malignancies and near-complete detection of aggressive Luminal B and triple-negative subtypes further support DBT as a valuable adjunct to 2D DM for ACR C and D populations in settings with appropriate subspecialty infrastructure. Because this cohort was single-reader and 100% biopsy-proven, these findings should be regarded as hypothesis-generating; prospective, multi-reader, multicenter validation in unselected screening populations, together with cost-effectiveness data, is needed before implementation-level recommendations can be made.

## Figures and Tables

**Table 1:** Patient and lesion characteristics (N = 123).

Characteristic	n	%
Total Patients	123	100
Age, mean ± SD (range)	46.7 ± 9.9 yr	27–82
**ACR Breast Density**		
Type C—Heterogeneously Dense	67	54.5
Type D—Extremely Dense	56	45.5
**Histopathological Outcome**		
Malignant	78	63.4
Invasive Ductal Carcinoma	62	79.5 *
Invasive Lobular Carcinoma	11	14.1 *
Other (Mucinous, Tubular, DCIS, Spindle Cell)	5	6.4 *
High-Risk (Radial Scar, ADH, Papilloma)	8	6.5
Benign (Fibroadenoma, Adenosis, UDH, etc.)	37	30.1
**Lesion Morphology**		
Mass only	54	43.9
Architectural Distortion (pure or mixed)	65	52.8
Asymmetry only	4	3.3
**Histological Grade (malignant, n = 76)**		
Grade 1—Low	20	26.3
Grade 2—Intermediate	35	46.1
Grade 3—High	21	27.6
**Molecular Subtype (malignant, n = 76 ^†^** **)**		
Luminal A	26	34.2
Luminal B	38	50.0
Triple Negative (TNBC)	10	13.2
HER2-Enriched	2	2.6

Note. ACR = American College of Radiology; ADH = atypical ductal hyperplasia; DCIS = ductal carcinoma in situ; UDH = usual ductal hyperplasia; TNBC = triple-negative breast cancer; HER2 = human epidermal growth factor receptor 2. * Percentage of malignant subgroup (n = 78). ^†^ Two cases within the malignant group (n = 78) lacked complete immunohistochemistry panels and were excluded from molecular subtype and histological grade analysis; accordingly, n = 76 for all grade and subtype data reported in the Results section.

**Table 2:** Comparative diagnostic performance: 2D mammography versus 2D + DBT (N = 123).

Metric	2D DM	2D + DBT	Δ	*p*-Value
Sensitivity	69.2% (58.3–78.4)	97.4% (91.1–99.3)	+28.2%	<0.001 *
Specificity	71.1% (56.6–82.3)	60.0% (45.5–73.0)	−11.1%	0.21
PPV	80.6% (69.6–88.3)	80.9% (71.7–87.5)	+0.3%	0.94
NPV	57.1% (44.1–69.2)	93.1% (78.0–98.1)	+36.0%	<0.01 *
Mean Visibility Score (0–5)	2.94 ± 1.19	4.52 ± 0.78	+1.58	<0.0001 ^†^
AUC (ROC)	0.727	0.922	+0.195	<0.001 ^‡^

Note. Values in parentheses indicate 95% Wilson score confidence intervals. 2D DM = two-dimensional digital mammography; DBT = digital breast tomosynthesis; PPV = positive predictive value; NPV = negative predictive value; AUC = area under the receiver operating characteristic curve; NS = not significant. * McNemar’s exact binomial test for paired proportions. ^†^ Wilcoxon signed-rank test for paired continuous/ordinal data. ^‡^ DeLong’s method for correlated ROC curves.

**Table 3:** Tumor biology, molecular subtype sensitivity, and architectural distortion performance.

Subgroup	n	2D DM Vis	DBT Vis	Δ (Gain)	*p*-Value
**Histological Grade**					
Grade 1 (Low)	20	2.75 ± 1.33	4.65 ± 0.59	+1.90	<0.001
Grade 2 (Intermediate)	35	2.74 ± 0.92	4.43 ± 0.65	+1.69	<0.001
Grade 3 (High)	21	3.38 ± 0.92	4.52 ± 0.60	+1.14	0.003
Spearman r (Grade vs. 2D vis)	—	r = +0.208	*p* = 0.071	—	NS
Spearman r (Grade vs. DBT vis)	—	r = −0.084	*p* = 0.471	—	NS
**Ki67 Proliferation Index**					
Low Ki67 (<20%)	27	2.88 ± 1.05	4.63 ± 0.63	+1.75	<0.001
High Ki67 (≥20%)	49	3.01 ± 0.98	4.45 ± 0.61	+1.44	<0.001
Mann–Whitney (Low vs. High DBT)	—	—	—	*p* = 0.151	NS
**Molecular Subtype—Sensitivity**					
Luminal A	26	69.2% (50.0–83.5)	96.2% (81.1–99.3)	+27.0%	<0.01
Luminal B	38	68.4% (52.5–80.9)	97.4% (86.5–99.5)	+29.0%	<0.001
Triple Negative (TNBC)	10	70.0% (39.7–89.2)	100% (72.2–100)	+30.0%	0.06 ^§^
**Architectural Distortion (AD)**					
AD-containing cases (all)	65	2.63 ± 1.07	4.54 ± 0.65	+1.95	<0.0001
AD malignant sensitivity	52	69.2%	100%	+30.8%	<0.0001
AD delta vs. Mass-only delta	—	—	—	Mann–Whitney	<0.0001

Note. Visibility scores are expressed as mean ± SD on the 0–5 Likert scale (malignant cohort only). Sensitivity values show 95% Wilson score confidence intervals. 2D DM = two-dimensional digital mammography; DBT = digital breast tomosynthesis; TNBC = triple-negative breast cancer; AD = architectural distortion; NS = not significant; Vis = visibility. ^†^ Wilcoxon signed-rank test for paired continuous/ordinal comparison. * McNemar’s exact test. ^§^ Fisher’s exact test due to small sample size (n = 10).

**Table 4:** Clinical decision change analysis: major upgrades, downgrades, and BI-RADS 0 resolution (N = 123).

Category	n	%
Major Upgrade (BI-RADS ≤ 3 on 2D → BI-RADS ≥ 4A on DBT)	27	22.0% of total
Confirmed Malignant on Histopathology	22	81.5% of upgrades
Confirmed High-Risk on Histopathology	2	7.4% of upgrades
Benign (False-Positive Upgrade)	3	11.1% of upgrades
**Upgrade PPV (Malignant + High-Risk/Total)**	24/27	88.9%
Major Downgrade (BI-RADS ≥ 4 on 2D → BI-RADS ≤ 3 on DBT)	0	0%
Total Decision Changes (any BI-RADS shift between sessions)	76	61.8% of total
**BI-RADS 0 on 2D DM → Resolved by DBT**	8/8	100%
Reclassified to BI-RADS ≥ 4 (all malignant)	7	87.5%
Reclassified to BI-RADS 3 (benign on biopsy)	1	12.5%
**McNemar Exact Test (sensitivity, 2D vs. DBT)**	—	*p* < 0.000001

Note. BI-RADS = Breast Imaging-Reporting and Data System; 2D DM = two-dimensional digital mammography; DBT = digital breast tomosynthesis; PPV = positive predictive value. “Major upgrade” specifically denotes a management-altering shift from a non-biopsy recommendation (BI-RADS 1–3) to a biopsy recommendation (BI-RADS 4A–5) facilitated directly by DBT interpretation.

**Table 5:** Tumor staging, surgical, and treatment characteristics in the definitive-surgery subset (n = 49/78 malignant cases operated at our institution).

Variable	n (%)
**Pathological T stage**	
T1	22 (44.9%)
T2	15 (30.6%)
T3	3 (6.1%)
T4	1 (2.0%)
ypT0 (pathologic complete response after NAC)	8 (16.3%)
**Nodal status**	
N0 (node-negative)	26 (53.1%)
N+ (N1–N3)	21 (42.9%)
Not assessable	2 (4.1%)
Multifocal disease	15 (30.6%)
Multicentric disease	3 (6.1%)
Lymphovascular invasion	1 (2.0%)
**Surgical procedure**	
Mastectomy	31 (63.3%)
Breast-conserving surgery	18 (36.7%)
Neoadjuvant chemotherapy received	27 (55.1%)
Pathologic complete response (Miller–Payne 5)	8/24 (33.3%)

Note. Data are presented for the subset of 49 malignant cases definitively operated at our institution (n = 49). Percentages reflect proportions within this subset. NAC = neoadjuvant chemotherapy; ypT0 = pathologic complete response in primary tumor. Two patients were classified as Nx (nodal status not assessable).

## Data Availability

The datasets generated and/or analyzed during the current study are not publicly available due to patient privacy and confidentiality agreements but are available from the corresponding author upon reasonable request.

## Abbreviations

**2D DM**	Two-Dimensional Digital Mammography
**ACR**	American College of Radiology
**AD**	Architectural Distortion
**AUC**	Area Under the Curve
**BI-RADS**	Breast Imaging-Reporting and Data System
**CI**	Confidence Interval
**DBT**	Digital Breast Tomosynthesis
**DCIS**	Ductal Carcinoma In Situ
**ER**	Estrogen Receptor
**HER2**	Human Epidermal Growth Factor Receptor 2
**IHC**	Immunohistochemistry
**NPV**	Negative Predictive Value
**PPV**	Positive Predictive Value
**PR**	Progesterone Receptor
**ROC**	Receiver Operating Characteristic (curve)
**SD**	Standard Deviation
**TNBC**	Triple-Negative Breast Cancer
